# Psychopathological intersection between obsessive-compulsive disorder and post-traumatic stress disorder: scoping review of similarities and differences

**DOI:** 10.47626/2237-6089-2021-0370

**Published:** 2023-05-19

**Authors:** Ygor Arzeno Ferrão, Rodrigo Bolsson Radins, João Vítor Bueno Ferrão

**Affiliations:** 1 Universidade Federal de Ciências da Saúde de Porto Alegre Porto Alegre RS Brazil Universidade Federal de Ciências da Saúde de Porto Alegre (UFCSPA), Porto Alegre, RS, Brazil.; 2 Consórcio de Pesquisa em Transtornos do Espectro Obsessivo-Compulsivo do Rio Grande do Sul Porto Alegre RS Brazil Consórcio de Pesquisa em Transtornos do Espectro Obsessivo-Compulsivo do Rio Grande do Sul, Porto Alegre, RS, Brazil.; 3 Programa de Pós-Graduação em Ciências da Saúde UFCSPA Porto Alegre RS Brazil Programa de Pós-Graduação em Ciências da Saúde, UFCSPA, Porto Alegre, RS, Brazil.; 4 Pontifícia Universidade Católica do Rio Grande do Sul Porto Alegre RS Brazil Pontifícia Universidade Católica do Rio Grande do Sul, Porto Alegre, RS, Brazil.

**Keywords:** Post-traumatic stress disorder, obsessive-compulsive disorder, psychopathology, scoping review

## Abstract

**Introduction:**

Although post-traumatic stress disorder and obsessive-compulsive disorder have distinct diagnostic criteria, some psychopathological phenomena seem to be shared, which may lead to misdiagnosis and erroneous treatment decisions. This scoping review explores the psychopathological similarities and differences between these two disorders.

**Method:**

The review complies with the recommendations of the Preferred Reporting Items for Systematic Reviews and Meta-Analyses (PRISMA) and included articles published in Portuguese, English, or Spanish in the last 50 years indexed in the PubMed database. Case-reports were excluded.

**Results:**

Fifty-three studies with different designs were included (30 [56.5%] were cross-sectional studies; eight [15.1%] were case-control studies; one [1.9%] was a cohort study; three [5.7%] were clinical trials; nine [17%] were reviews/systematic reviews; and two [3.8%] were meta-analyses). The main psychopathological aspects described by the studies included were flashbacks x obsessions; avoidant behavior (AB); depressive, anxious, and somatic symptoms; sexuality, sleep, and appetite; psychiatric comorbidities; and suicidality. The intersection between clinical features seems to occur in the extrinsic psychopathological dimension.

**Conclusion:**

The disorders’ core psychopathological symptoms (intrinsic characteristics) are distinctly different, since flashbacks and obsessions are consequences of different predominant defective mental functions: the former derives from defective memory, the latter from defective thought. Along the same lines, the ABs observed in the two disorders are products of different purposes and inner necessities.

## Introduction

Although post-traumatic stress disorder (PTSD) and obsessive-compulsive disorder (OCD) have distinct diagnostic criteria according to the DSM-5,^1^ they seem to share some psychopathological phenomena, which could lead to misdiagnosis and, thus, to erroneous treatment decisions.^2^ For example, Green^3^ reported that anxiety, insomnia, distressing and recurrent dreams, flashback imagery, intrusive thoughts, irritability, poor concentration, avoidant behavior (AB), and detachment were present for 70% of PTSD patients. While some of the abovementioned symptoms may also be present for OCD patients (anxiety, insomnia, intrusive thoughts, irritability, poor concentration, and ABs), others, such as flashbacks, are not always easily distinguished from obsessions. To make the comparison even more complex, patients with OCD report increased frequency and severity of stressful life events (SLE) (including traumatic events) in the 6 months prior to the onset of symptoms,^4^ and close to 60% of OCD patients mention some SLE, making their occurrence a risk factor for comorbidity of PTSD with OCD.^5^

**Figure 1:**
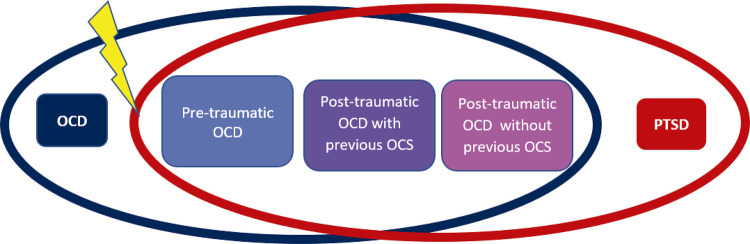
Illustration of a possible psychopathological intersection continuum between obsessive-compulsive disorder (OCD) (dark blue circle) and post-traumatic stress disorder (PTSD) (red circle). After a traumatic event (yellow bolt), three situations may occur: 1) PTSD in patients with pre-traumatic OCD (real comorbidity) (light blue square); 2) PTSD in patients with previous obsessive-compulsive symptoms (OCS) (purple square); and 3) post-traumatic OCD (pink square). Neurobiologically, post-traumatic OCD may be linked to PTSD (adapted from Fontenelle et al.10).

Trying to understand the psychopathological overlap in PTSD-OCD comorbidity, Auxéméry^6^ postulates that when PTSD patients re-experience, they become anxious; that hyper arousal increases anxious reactivity; and that avoidance strategies may increase anticipatory anxiety, reinforcing the existence of a “post-traumatic OCD” and also of many other “post-traumatic anxiety disorders.” Rather than OCD, Freud would probably have used the term “obsessional neuroses,” since after a traumatic event (such as, for example, a patient who heard from a fellow army officer a description of a Chinese torture method in which a large pot containing a live rat was strapped to the buttocks of the victim, and the rat, encouraged by a red-hot poker, would gnaw its way out through the victim’s anus), patients would exacerbate some defense mechanisms, such as rationalization, doubt, undoing, and displacement.^7^ However, Lafleur et al.^8^ did not find a relationship between the specific type of OCD symptoms exhibited and a history of psychologically traumatic events in children with OCD. It has also been argued that the severity of the traumatic event could predict obsessive-compulsive symptoms (OCS).^9^ More recently, after reporting that 19.1% of 1,001 OCD patients also had PTSD, Fontenelle et al.^10^ elegantly proposed a continuum of distinct phenotypes of OCS in this context, ranging from pure OCD, passing through pre-traumatic OCD, post-traumatic OCD with previous OCS, and post-traumatic OCD without previous OCS, as can be seen in the adapted Figure 1.^10^ On the other hand, other authors found no relation between traumatization or PTSD and OCD when compared to healthy controls.^11^

Besides the possibility of misdiagnosis, the relevance of the intersection between PTSD and OCD lies in the fact that some specific aspects of the comorbidity could interfere with treatment response or adherence,^12^ such as the association with a lower level of insight,^13^ higher levels of OCS severity,^10^ higher suicidality,^10,14-16^ and higher prevalence of psychotic features,^17^ including dissociation.^18-21^ However, unexpectedly, Shavitt et al.^22^ found that OCD patients with PTSD exhibited a greater magnitude of response when compared with OCD patients without PTSD in specific OCD symptom dimensions.

Although other literature reviews have been carried out on this comorbidity, none has comprehensively explored the intersection between the psychopathological aspects of the two conditions, with a special focus on differentiating between symptoms that aid in differential diagnosis in a didactic manner. Thus, the main objective of this review is to explore the psychopathological similarities and differences between the two disorders, specifically comparing aspects such as flashbacks and obsessions; the occurrence of ABs; the presence of depressive, anxious, and somatic symptoms; sleep pattern; appetite; sexuality; psychiatric comorbidities, and suicidality, with the intention of providing a basis for future neurobiological and therapeutic studies.

## Methods

**Figure 2:**
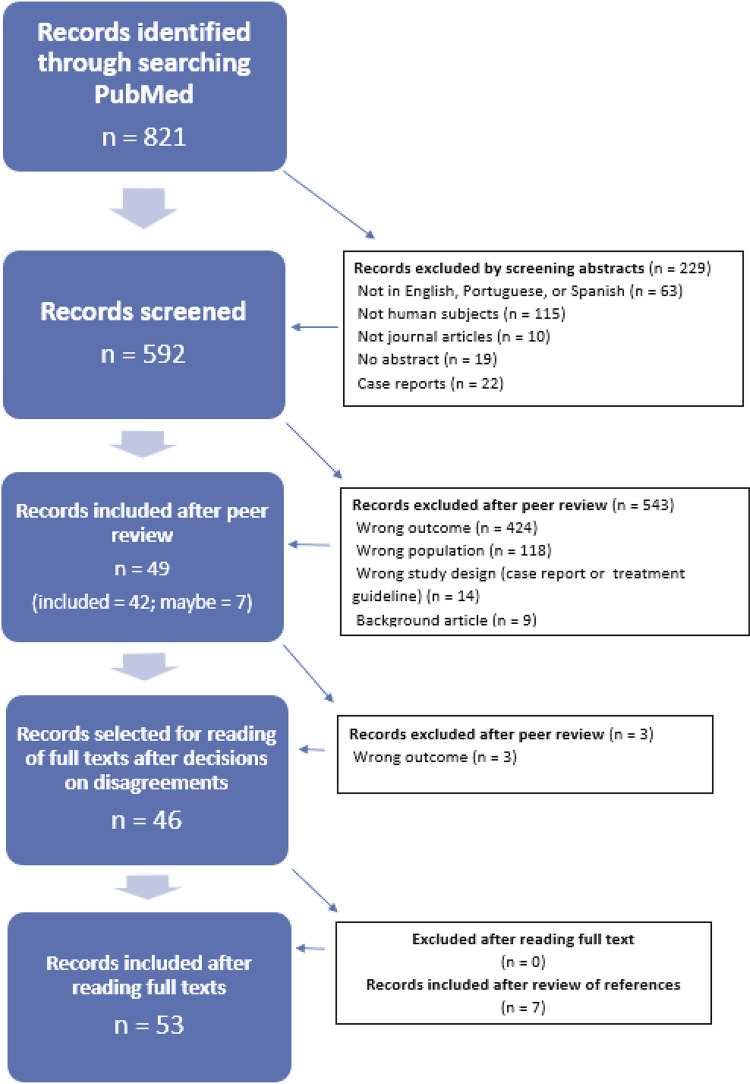
Flowchart illustrating study selection according to the Preferred Reporting Items for Systematic Reviews and Meta-Analyses (PRISMA)

This scoping review complies with the recommendations of the Preferred Reporting Items for Systematic Reviews and Meta-Analyses (PRISMA)^23^ and was based on the following Boolean searches: “Obsessive-Compulsive Disorder AND Post-Traumatic Stress Disorder,” “Obsessive-Compulsive Disorder AND trauma,” “obsession AND Post-Traumatic Stress Disorder,” “obsession AND trauma,” “obsession AND flashback,” and “Obsessive-Compulsive Disorder AND flashback.” The inclusion criteria were: journal articles published in Portuguese, English, or Spanish, in the last 50 years (1971-2021), indexed on the PubMed database, with abstracts available, and involving humans. Case-studies were excluded. Literature searches and selection were conducted by three researchers, two of them acting as independent and blinded reviewers (RR and JVBF). First, a search was conducted on the PubMed database. All articles and abstracts initially retrieved (n = 592) were analyzed and those that were appropriate were selected. Both reviewers used the same inclusion and exclusion criteria mentioned above and compared their results with one another. Any disagreements that occurred during the initial process of analysis and selection were resolved by a third researcher (YAF). While reading the full text of the remaining papers, the authors searched for the following: any definitions or psychopathological features of the terms “obsession” and “flashbacks”; presence and description of AB; any clinical aspect or mention of depressive, anxious, or somatic symptoms; any mention of specific features of sleep, appetite, or sexuality due to the core psychopathological aspects of the disorders (but not due to the treatment); the main psychiatric comorbidities of both disorders; and any descriptive aspect of suicidality. The references of the papers selected were also reviewed. The results of the search are illustrated in Figure 2. No restrictions were established for the references the authors used to help them formulate the discussion section. Table S1 (online-only supplementary material) lists details of the search results.

## Results

Fifty-three studies with a variety of research designs were included in this psychopathological review: 30 (56.5%) were cross-sectional studies; eight (15.1%) were case-control studies; one (1.9%) was a cohort study; three (5.7%) were clinical trials; nine (17%) were reviews/systematic reviews; and two (3.8%) were meta-analyses. Table 1 summarizes the studies reviewed, indicating each study’s research design, population, and sample size or number of studies or references reviewed, and the OCD and PTSD instruments used to investigate the main clinical symptoms of both disorders. The main results are presented and discussed below Table 1, but, to summarize, the main psychopathological aspects of OCD and/or PTSD aspects described in the studies were as follows: only five papers compared the phenomenology of the intersection between the concepts of flashbacks and obsessions; the occurrence of AB in both disorders was investigated in only one study; four authors studied the concomitant depressive symptoms in both disorders; the concomitant anxious and somatic symptoms in both disorders were covered in 19 studies; aspects of sleep (six papers), appetite (four papers), and sexuality patterns (nine papers) have not yet been compared directly between OCD and PTSD in the literature; and the most common psychiatric comorbidities of each disorder were described in 14 studies, including suicidality (specifically in 12 studies), which was considered a transdiagnostic phenomenon for the purpose of this study.

**Table 1:** Summary of the 53 studies selected for inclusion in the review

Study	Study design	Population (n)	Specific OCD/PTSD instruments used
Afari^24^	Meta-analysis	71 studies	-
Auxéméry^6^	Review	89 references	-
Avdibegovic^25^	Cross-sectional	PTSD (n = 217)	HTQ
Ay^15^	Cross-sectional	OCD (n = 67)	Y-BOCS, CTQ
Badour^26^	Cross-sectional	PTSD (n = 49)	PDS, OCI-R
Barzilay^17^	Cross-sectional	Healthy youths (n = 7,054)	Kiddie SADS
Bond^27^	Cross-sectional	Spouses (n = 31) and close relatives (n = 25) of hospitalized patients with acute burns	MPSS-SR
Brady^28^	Review	77 references	-
Brakoulias^29^	Cross-sectional	OCD (n = 3,711)	Y-BOCS, SCID-I
Brewin^30^	Review	51 references	--
Brewin^31^	Review	47 references	-
Ferrão^32^	Case-control	OCD (n = 1,001)	DY-BOCS, Y-BOCS, SCID-I
Fontenelle^21^	Case-control	OCD (n = 34)	THQ, DES, OCI
Fontenelle^10^	Case-control	OCD (106 patients with OCD after PTSD [post-traumatic OCD], 41 patients with OCD before PTSD [pre-traumatic OCD], and 810 OCD patients without PTSD [non-traumatic OCD])	DY-BOCS, Y-BOCS, SCID-I
Forbes^33^	Cross-sectional	PTSD (n = 835)	DSM-IV criteria
Fostick^34^	Cross-sectional	Veterans	DSM-IV criteria
Franklin^2^	Case-control	Veterans	DOCS, PCL-5
Gershuny^35^	Open clinical trial	OCD (n = 15)	DSM-IV criteria
Gershuny^12^	Cross-sectional	OCD (n = 104)	Naturalistic retrospective chart reviews of pre-treatment clinical intake files
Grabe^11^	Cross-sectional	OCD (n = 210), controls (n = 133)	German version of the SADS - Lifetime Version for Anxiety Disorders (DSM-IV)
Gros^36^	Cross-sectional	Veterans (n = 854)	MINI, PDS
Huppert^37^	Case-control	OCD (n = 128), PTSD (n = 109), other anxiety disorders (n = 63), college students (n = 40 )	R-OCI, PDS
Iverach^38^	Review	231 references	-
Kessler^39^	Cross-sectional	National Comorbidity Survey (n = 5,877 persons)	DSM-III-R PTSD module from the Diagnostic Interview Schedule and the CIDI
Khosravani^16^	Case-control	OCD (n = 70) and controls (n = 60)	CTQ-SF, Y-BOCS, DOCS
Kroska^40^	Cross-sectional	At-risk adolescents (n = 51) and a group of college students (n = 400)	ETI-SR-SF, AFQ-Y , R-OCI
Lafleur^8^	Cross-sectional	OCD (n = 278)	CY-BOCS, Kiddie SADS-E (Epidemiological Version)
Liu^41^	Cross-sectional design with gene-environment interaction	PTSD (n = 1,131)	PCL-5
Lochner^19^	Case-control	OCD (n = 110)	DES, CTQ
Lochner^20^	Cross-sectional	OCD (n = 83)	DES, CTQ
Mataix^42^	Review (editorial)	6 references	-
Menzies^43^	Cross-sectional	OCD (n = 171)	MFDS, VOCI, C-LFDS
Menzies^44^	Cross-sectional	OCD (n = 98)	MFDS, VOCI, C-LFDS
Menzies^45^	Cross-sectional	OCD (n = 79)	MFDS, VOCI, C-LFDS
Merrill^46^	Cross-sectional	OCD (n = 104)	Naturalistic retrospective chart reviews of pre-treatment clinical intake files
Miller^47^	Meta-analysis	24 studies	-
Morina^9^	Cross-sectional	PTSD (n = 51)	R-OCI, PDS
Nacasch^48^	Cross-sectional	PTSD (n = 44)	SCID
Nissen^49^	Cross-sectional	OCD (n = 317)	Medical records
Ojserkis^50^	Cohort	OCD (n = 266)	YBOCS, SCID, DSM-IV
Pellegrini^51^	Systematic review	61 studies	-
Richards^52^	Review	290 references	-
Ruscio^53^	Cross-sectional	National Comorbidity Survey (n = 2,073 persons)	DSM-IV
Semiz^18^	Cross-sectional	OCD (n = 120)	YBOCS, TEC, DES
Shavitt^22^	Randomized clinical trial	OCD (n = 219)	YBOCS, DYBOCS
Sikharulidze^54^	Cross-sectional	Veterans (n = 2,799)	PCL-5
Stein^55^	Review	171 references	-
Torres^14^	Cross-sectional	OCD (n = 582)	DY-BOCS, Y-BOCS, SCID-I.
Torres^56^	Cross-sectional	OCD (n = 955)	DY-BOCS, Y-BOCS, SCID-I
Torresan^57^	Cross-sectional	OCD (n = 858)	DY-BOCS, SCID-I
Unseld^58^	Cross-sectional	Adult cancer patients (n = 1,017)	PTSS-10
Valderrama^59^	Case-control	OCD (n = 605)	
Wheaton^60^	Randomized controlled trial	OCD (EX/RP [n = 40]), risperidone (n = 40), and placebo (n = 20))	SCID-I

Population studied and instruments used: only one (2.4%) of the 42 studies that included patients/subjects directly compared obsessive-compulsive disorder (OCD) and post-traumatic stress disorder (PTSD) patients; 25 (29.5%) included OCD patients; six (14.3%) included PTSD patients; four (9.5%) included war veterans (whom authors infer to have PTSD); and six (14.3%) investigated healthy specific or community volunteers.

EX/RP = exposition/response prevention; n = sample size.

Non-specific instruments: AFQ-Y = Avoidance and Fusion Questionnaire-Youth; CIDI = Composite International Diagnostic Interview; C-LFDS = Collett-Lester Fear of Death Scale; DSM-IV = lifetime Diagnostic and Statistical Manual of Mental Disorders, 4th ed.; Kiddie SADS-E = Kiddie Schedule for Affective Disorders and Schizophrenia (Epidemiological Version); MFDS = Multidimensional Fear of Death Scale; MINI = Mini International Neuropsychiatric Interview; SADS = Schedule for Affective Disorders and Schizophrenia; SCID-I = Structured Clinical Interview for DSM-IV Axis I disorders.

Obsessive-compulsive disorder instruments: CY-BOCS = the Children’s Yale-Brown Obsessive Compulsive Scale; DOCS = the Dimensional Obsessive-Compulsive Scale; DY-BOCS = Dimensional Yale-Brown Obsessive-Compulsive Scale; R-OCI or OCI = Revised Obsessive-Compulsive Inventory Scale; VOCI = Vancouver Obsessive Compulsive Inventory subscale; Y-BOCS = the Yale-Brown Obsessive Compulsive Scale.

Post-traumatic stress disorder instruments: CTQ = Childhood Trauma Questionnaire; CTQ-SF = Childhood Trauma Questionnaire-Short Form; DES = dissociative experience scale; ETI-SR-SF = Early Trauma Inventory Self-Report Short Form; HTQ = Harvard Trauma Questionnaire; MPSS-SR = Modified PTSD Symptom Scale; PCL-5 = PTSD Checklist for DSM-5; PDS = the Posttraumatic Stress Diagnostic Scale; TEC = Traumatic Experiences Checklist; THQ = Trauma History Questionnaire.

The OCD and PTSD specific instruments most used were (see Table 1 for further details): 1) for OCD: Y-BOCS (Yale-Brown Obsessive Compulsive Scale); DY-BOCS (Dimensional Yale-Brown Obsessive-Compulsive Scale); DOCS (Dimensional Obsessive-Compulsive Scale); and R-OCI or OCI (Revised Obsessive-Compulsive Inventory Scale); 2) for PTSD: PDS (Posttraumatic Stress Diagnostic Scale); DES (Dissociative experience scale); and CTQ or CTQ-SF (Childhood Trauma Questionnaire, full version or Short Form; and PCL-5: PTSD Checklist for DSM-5; 3) Non-specific instruments (used to diagnose or to confirm diagnoses): SCID-I (Structured Clinical Interview for DSM-IV Axis I disorders); and SADS (Schedule for Affective Disorders and Schizophrenia, adult or Kiddie forms; and other psychometric instruments: MFDS (Multidimensional Fear of Death Scale); C-LFDS (Collett-Lester Fear of Death Scale). The DY-BOCS was only used by Brazilian authors, and only one team of authors used the MFDS and C-LFDS (in three different papers).

## Discussion

Although only one paper directly compared OCD and PTSD patients,^37^ most of the authors used one group of patients (OCD or PTSD) or “at-risk healthy subjects” to investigate the occurrence of symptoms (using validated instruments) specific to the other diagnostic group. Thus, it was also necessary to select those papers to help to understand the scoping concepts considered as possible confounding issues in differential diagnosis between OCD and PTSD.

### Psychopathological intersection between OCD and PTSD

#### Flashbacks and obsessions

Although conceptually easy to distinguish, both phenomena share some features, which may, in fact, contribute to some patients finding it difficult to differentiate between them on self-report measures.^2^

**Flashbacks.** Pierre Janet described how memories of traumatic experiences are dissociated from normal consciousness, resulting in powerful and uncontrollable re-enactments of the events.^61^ The most striking characteristics of this traumatic re-experiencing in PTSD are its involuntary and uncontrollable nature, the strong sensory impressions, and the sense of “nowness,” or of the event occurring in the present.^31^ The DSM-5 and the proposed ICD-11 PTSD criteria have adopted a definition in which flashbacks are seen as existing along a continuum between extreme episodes (in which individuals even lose contact with their surrounding environment for periods of minutes or more) and any intrusive memories that are accompanied by a sense of reliving the event in the present.^1,31,62^ Some patients describe flashbacks as “a type of memory that you experience as markedly different from those memories of the event that you can retrieve at will, and in some cases, leading even to distorted time perception.”^31^ Currently, there is enough evidence to support the position that flashbacks involve a perceptual memory system, which is distinct from ordinary episodic memory.^31^ While vivid memories are associated with additional activity in medial temporal lobe structures (such as the hippocampus), dual representation theory predicts that flashbacks may be associated with increased activity in motor areas and the insula and amygdala, but reduced activity in the medial temporal lobe.^31,63^

**Obsessions.** Esquirol described OCD as a disorder in which the sufferer is “chained to actions that neither reason nor emotion have originated, that conscience rejects, and will cannot suppress.”^64^ Pierre Janet described how obsessions and compulsions develop over three phases: initially characterized by a “psychasthenic” state (indecisiveness, need for perfectionism and orderliness, and restricted emotional expression); followed by a stage of “forced agitations” (need for symmetry, repeating, and checking); and, finally, manifestations of frank obsessions and compulsions (aggressive, religious, and sexual themes).^65^ Whereas flashbacks are classified as a memory disorder, the DSM-5 and ICD-11 define an obsession as a thought disorder: “recurrent and persistent thoughts, images, urges or impulses that are experienced as intrusive and unwanted, and that in most individuals cause marked anxiety or distress.”^1,62^ Obsessions may have any universal human content, including aggressive/violence/catastrophes, contamination, sexual/moral/religious, symmetry/order/arrangement, hoarding and many others.^65^ Rachman^64^ postulates that obsessions are caused by catastrophic misinterpretations of the significance of one’s unwanted intrusive thoughts. Certain considerations are needed to fully understand the factors that precipitate intrusive thoughts and those linked to vulnerability to them.^66^ Vulnerability may be due to dysregulation of one or more than one of the following neurocircuits: 1) the fronto-limbic circuit which is involved in producing emotional responses, such as fear and anxiety; 2) the sensorimotor circuit which produces and controls motor behavior and integration of sensory information; 3) the ventral cognitive circuit which is involved in self-regulatory behavioral control; 4) the ventral affective circuit which processes and responds to reward; and 5) the dorsal cognitive circuit which mediates executive functions (e.g., working memory, planning) and regulation of emotion.^66-68^ Since it can be difficult to dissociate behavior and cognition in clinical practice (one leads to the other and vice-versa), we decided to describe all five circuits here, even when identifying that some circuits seem to be more behavioral (compulsive) than cognitive (obsessive).

Thus, Table 2 attempts to summarize the most prevalent similarities and differences between psychopathological aspects of flashbacks and obsession. According to Fontenelle et al.,^10^ comorbidity between PTSD (flashbacks) and OCD (obsessions) is not uncommon, leading some of the features described below to be present in the same patient, at least for a period of time.

**Table 2:** Psychopathological clinical aspects of flashbacks and obsessions: similarities and differences

	Flashbacks	Obsessions
Related to a traumatic event^1,5,8,11,17,21,26,38,55,60,69-74^	+++	+
Involuntary/intrusive nature^1,2,6,11,24-30^	+++	+++
Uncontrollable/difficult to control nature^1,2,6,11,24-30^	+++	+++
Intermittent nature^1,2,6,11,24-30^	+++	+++
Sense of “nowness”^1-3,6,24-27^	+++	-
Loss of contact with the surrounding environment for periods of minutes or more^24-27^	+++	++
Distorted time perception^1,2,6,11,24-30^	+++	+
Interferes with attention/concentration^1,2,6,11,24-30^	+++	+++
Indecisiveness/doubt^1,11,24-37^	-	+++
Causes marked anxiety or distress^49-65^	+++	+++
Leads to somatic symptoms^62-65^	+++	+++
Recurrent and persistent^1,2,6,11,24-37,67,68^		
Thoughts	-	+++
Images	+++	+++
Sounds	+++	+
Smells	+++	-
Tastes	++	-
Urges	-	+++
Impulses	+++	+++
Occurs while sleeping^75-80^	+++	-
May become chronic^1,2,6,11,24-30^	+	+++
Egosyntonic (poor insight)^1,13,24-26^	+	+++
Genetic inheritance/familial^1,26,29^	-	+++
Neurocircuit dysfunctions^1^^,^^6^^,^^10^^,^^13^^,^^20^^,^^25-27^^,^^29^^,^^31^^,^^32^		
Motor areas (supplementary motor area)	+++	+++
Insula	+++	+++
Amygdala	+++	++
Medial temporal lobe	+++	+++
Prefrontal cortex	-	+++
Striatum (putamen and caudate)	-	+++
Thalamus	-	+++
Inferior frontal gyrus	-	++
Orbitofrontal cortex	-	+++
Nucleus accumbens	-	+++

+ = weakly related/low possibility/prevalence; ++ = moderately related/ moderate possibility/prevalence; +++ = strongly related/high possibility/prevalence.

#### Avoidant behavior

An AB can be understood as any voluntary act aimed at preventing real or imaginary contact with situations, places, objects, thoughts, or images perceived as dangerous and/or with the potential to activate anxiety or undesirable cognitive phenomena (thoughts or memories).^67^ Such ABs can be explained by the “Mowrer two-stage model”^68^: in a first stage, an initially neutral stimulus becomes an aversive stimulus due to successive pairings of that stimulus with unpleasant emotions (classic conditioning); in a second stage, the individual starts to avoid or rid themselves of the discomfort caused by the stimulus now conditioned. If performing the AB is followed by relief, the probability of repeating it increases (negative reinforcement or operant conditioning),^81-83^ turning it into a possible mechanism of the association between trauma and obsession-compulsion.^40^

According to the DSM-5, ABs are essential for a diagnosis of PTSD, but not for OCD.^1^ Forbes et al.^33^ postulated that requiring active avoidance helped to define the unique aspects of PTSD and reduced spurious diagnoses of PTSD in those with depression (reducing this comorbidity by 44%). Corroborating this, Liu et al.^41^ described a relation between the serotonin transporter gene-linked polymorphic region (5-HTTLPR), interacting with trauma exposure to increase general risk for symptoms of PTSD, including ABs. Meanwhile, AB is described in almost 70% of OCD patients^60^ and has been related to higher severity, to the contamination/cleaning subtype, and to a reduced probability of remission.^49^ Thus, it is unequivocal that AB may represent not only a structural, but also a learned psychopathological feature which interferes in the presentation and in the treatment response. For PTSD, the ABs seems to happen *a posteriori* (based on reasoning from known/real past traumatic facts or events rather than on assumptions or predictions), as an attempt to avoid triggering negative memories or feelings directly related to the event. Such an AB is easily understood as reasonable or rational by the patient or by anybody else. In contrast, in OCD, ABs occur to prevent suffering from imaginary or exaggerated harmful situations, places, objects, or thoughts. Sometimes, but not always, OCD patients perceive the motivation underlying the AB to be reasonable/rational, but other people almost always easily understand it as nonsense or even absurd and do not agree with the AB.

#### Depressive symptoms

Depressive symptoms are prevalent in patients with OCD and in patients with PTSD.^1^ Accordingly, major depressive disorder (MDD) is the most common comorbidity in OCD patients (up to 2/3 may experience a depressive episode during their lifetime).^1,84,85^ In PTSD, depressive symptoms are also quite common, since “negative mood” is part of the diagnostic criteria for this disorder (Criterion D in the DSM-5). It should be noted, however, that depressed mood in PTSD is associated with a traumatic event, but it can lead to distorted and negative cognitions in relation to the event, decreased interest in activities, and feelings of alienation or inability to feel positive emotions,^1^ mimicking major depression and making differential diagnosis complex and difficult to accomplish.^86^ We can speculate that major depression in OCD may occur due to the severity, chronicity, or even the specific content of the obsessions, which may lead patients to feel low self-esteem, guilt, or shame, since the content of some obsessions are not morally or socially acceptable (e.g., sexual, aggressive, blasphemous).

Gershuny et al.^12^ and Huppert et al.^37^ hypothesized that, in addition to the intersection with psychopathological symptoms, depressive symptoms could be a mediating factor in this comorbidity, since the rate of diagnosis of PTSD was higher in patients with OCD who also suffered from depression.^35,37^ Corroborating this hypothesis, in a study with 104 individuals who had this comorbidity, Merrill et al.^46^ found that depressed patients had higher levels of OCS and greater severity of post-traumatic stress symptoms. However, Morina et al.^9^ found that only the severity of the posttraumatic stress symptoms was predictive of OCS, whereas number of traumatic life event types and depressive symptoms were not. How depression may interconnect PTSD and OCD, however, needs further investigation.

#### Anxious and somatic symptoms

As with depressive symptoms, there is also a high prevalence of anxiety in both OCD and PTSD. The association with OCD is so clear that in the DSM-III, III-R, and IV it was considered to be one of the anxiety disorders and these symptoms were considered key to syndromic diagnosis.^55^ Despite the change in DSM-5, many experts questioned the decision, considering that anxiety is indeed a central aspect of the clinical presentation of OCD.^1,42^ An association also occurs in PTSD, since evidence shows that people who experience the disorder are at higher risk of anxiety symptoms.^27,44^ Even though the clinical presentation of PTSD can vary according to the severity of the symptoms and to the nature of the trauma experienced, anxiety is certainly a prevalent symptom. For example, 69% of the spouses of patients admitted to an intensive care unit (ICU) had clinically restricted anxiety.^87^

Iverach et al.^38^ emphasize that fear of death is part of the anxiety spectrum, but can be considered as a transdiagnostic symptom since it is present in several mental disorders. While this fear can lead to safer behaviors and the search for a more meaningful life, when intense or excessive it causes extreme anxiety and leads to maladaptive behaviors.^43^ In OCD, the fear of dying can be clear in some cases, as in the contamination dimension, for example, the fear of dying or of causing death because of being contaminated is what motivates suffering and repetitive behaviors. The same occurs in the aggressiveness dimension, where the fear of death is central to repetition of symptoms. One example of this is when patients over-check the windows and doors of the home, for fear that a burglar will invade the home and put their lives at risk.^88,89^ Anxiety related to the fear of dying seems to be more prevalent in some specific dimensions, such as contamination, checking, and hoarding, or in patients with obsessive predominance.^44^ Badour et al.^26^ demonstrated that the intensity of peritraumatic self-focused disgust was significantly related to contamination-based OC symptoms while peritraumatic fear and other-focused disgust were related to posttraumatic stress symptoms. Menzies et al.^45^ suggested in a study with 98 patients with OCD that the presence of fear of dying may increase the risk of comorbidity with anxiety disorders and influence the trajectory of the disorder.

In the clinical presentation of PTSD, autonomic and anxiety symptoms are also more the rule than the exception. Criterion B of DSM-5 mentions that there may be intrusive memories, distressing dreams or intense physiological reactions and Criterion E includes symptoms such as hypervigilance and exaggerated startle response.^1,44,90^ Such symptoms are clinically associated with anxiety, as well as with ABs that are also common in clinical practice and require special treatment in PTSD.^3^ Since anxiety symptoms are part of both OCD and PTSD and can also be a comorbid disorder, regular symptom monitoring is important, requiring individualized investigation.^44,90^

In addition to the correlation with anxiety, associations between PTSD and somatic syndromes have been reported in the literature. Exposure to traumatic events seems to be associated with a higher prevalence of fibromyalgia, chronic pain, and chronic fatigue syndrome.^24,91^ The DSM-5 description of the disorder itself explains that dissociative symptoms and flashbacks are so prevalent in the clinical condition that they are part of the diagnostic criteria.^1^ In addition, somatic complaints such as headache, body aches, and gastrointestinal symptoms tend to be more intense in individuals with PTSD than in those without this diagnosis.^25^ However, Sikharulidze et al.^54^ argue that this correlation is mediated by depressive and anxious symptoms, since it loses statistical significance when patients with PTSD are controlled for these symptoms. Although some somatic obsessions may be present in OCD, the evidence of the relationship between OCD and somatic disorders is not robust as with PTSD. Valderrama et al.^59^ even described, for example, that when a somatic symptom or disorder (such as body dysmorphic disorder, for example) occurs with OCD, PTSD would probably also be present, making it a predictor of presence of BDD symptoms among OCD individuals who have experienced at least one lifetime traumatic event. The absence of evidence is interesting, since most patients with OCD (60-70%) have sensory phenomena, which consist of physical sensations – often aversive – that lead to compulsive behavior.^32^ This phenomenon has a somatic characteristic, considering that patients actually “feel” the discomfort, even without a real stimulus of the same intensity.^32,92^ Thus, sensory phenomena could be misdiagnosed as “somatization,” but this seems not to occur. One possibility that could explain this is that, when a patient is diagnosed with OCD, clinicians may consider that somatization is part of the disorder and not a comorbidity. Feske^93^ and Otte^75^ speculate that somatization may also be mediated by depressive symptoms and be causally related to PTSD or OCD.

To further understand the relationship between anxiety and depression, Lee et al.^76^ examined the factor structure of the combined items from the Beck Anxiety Inventory (BAI) and the Beck Depression Inventory-II (BDI-II) in a psychiatric outpatient population. They suggested that the symptoms could be grouped into five factors: somatic anxiety, cognitive depression, somatic depression, subjective anxiety, and autonomic anxiety.^76^ Along the same lines, extrapolating their results, but to better understand how depressive and anxious symptoms can present in PTSD and/or OD, we could conjecture (but based on clinical experience) that depressive and anxious symptoms could be grouped into cognitive, somatic, autonomic, behavioral (including AB), and emotional (subjective) subgroups, which could help us to distinguish these clusters in PTSD and OCD patients, as summarized in Table 3. Nonetheless, this phenomenological hypothesis requires appropriate future investigation.

**Table 3:** Comparison of occurrence of depressive and anxious symptom subtypes in PTSD and OCD^48^-^65^,^69^-^74^,^81^-^83^

Depressive and anxious symptom clusters	PTSD	OCD
Cognitive	+++	+++
Somatic	+++	+
Autonomic	++	+++
Behavioral	++	+++
Emotional (subjective)	+++	++

+ = weakly related/low possibility/prevalence; ++ = moderately related/moderate possibility/prevalence; +++ = strongly related/high possibility/prevalence; OCD = obsessive-compulsive disorder; PTSD = post-traumatic stress disorder.

#### Sexuality, appetite, sleep pattern

Obsessive compulsive disorder and PTSD may both interfere in sexuality, appetite, and sleep patterns. Sexuality is often ignored in clinical practice because the therapeutic focus is on symptom reduction, but it has gained attention in the recent literature on the subject.^77^ Evidence shows that individuals with OCD tend to suffer from more dysfunction and less sexual satisfaction than the general population, which contributes to worse quality of life.^78,79^ Many factors may be involved in this relationship, but Pozza et al.^92^ pointed out that patients with the dimension of contamination or excessive sensitivity to disgust seem to be more prone to sexual inhibition, due to anxiety related to imaginary risks. In addition, the DSM-5 emphasizes that in the sexuality dimension, patients may present obsessions, mental images of sexual characteristics that cause suffering, which leads to a relationship with impaired sexuality.^1^ When depressive symptoms are associated with the condition, impairment of sexual quality of life is expected, since sexual symptoms also occur in MDD.^94^ OCD also seems to be associated with the compulsive sexual disorder proposed in the ICD-11,^62^ which can also contribute to low sexual satisfaction.^95^ Evidence also points to higher levels of sexual dissatisfaction and dysfunction in PTSD patients.^96,97^ It is possible that when the traumatic PTSD event is of a sexual nature, this association is even more prevalent, considering that intrusive memories, flashbacks, and negative cognitions are causally related to the event, harming future relationships.^98^

Behavioral changes associated with eating occur in many disorders, most of the time causing higher rates of obesity or overweight. However, OCD does not appear to increase this risk, although evidence on the topic is still scarce.^99^ On the other hand, there is much evidence in the literature that PTSD is indeed correlated with obesity and a higher risk of metabolic syndrome.^69,100-102^ It is also possible to speculate that weight gain, obesity, or metabolic syndrome may occur in PTSD or OCD as a consequence of psychopharmacological therapies, since most of the medicines used (antidepressants, mood stabilizers and antipsychotics) seem to increase appetite.^70^

Sleep disorders are prevalent in PTSD, but in addition to occurring after the traumatic event, they also seem to be considered predictive of development of the disorder.^52,101,102^ Like nightmares, insomnia seems to be an important symptom of PTSD, given that cognitive behavioral therapy aimed at reducing insomnia also reduces PTSD symptoms.^103^ OCD patients also present marked sleep impairment, with severity varying according to the severity of the clinical condition. The type of sleep impairment varies between patients, from reduction in total sleep time or sleep efficiency to delayed specific sleep phases and impairment seems more frequent in patients with more depressive symptoms.^104,105^

#### Psychiatric comorbidities

According to the American Psychiatric Association (APA), approximately 3.5% of American adults are affected by PTSD every year, and it is estimated that one in 11 will have a lifetime diagnosis of PTSD.^1,28,39^ The lifetime prevalence of OCD is estimated to be around 1.5%.^71^ However, “pure” occurrence is not the rule with either disorder; PTSD has a high rate of comorbidity: 16% of patients have one psychiatric comorbidity, 17% have two, and 50% have three or more; OCD also has a high rate of psychiatric comorbidities, which can exceed 90% of patients.^53,56,72-74^ Since a psychiatric comorbidity may determine a specific psychopathological presentation and can influence the conventional treatment response, we present the most common comorbidities in PTSD and OCD in a comparison table (Table 4). As can be seen, the most common groups of psychiatric comorbidities for both PTSD and OCD are affective disorders (almost 50 and 68%, respectively), anxiety disorders (almost 55 and 75%, respectively), and substance use/abuse/dependence (almost 78 and 36%, respectively),^28,50,73,106^ although prevalence rates differ between the two disorders. Differing values between studies for each disorder may reflect different methodological aspects and discrepancies between recruitment strategies employed at primary and tertiary services. For example, Torresan et al.^57^ found some differences between male and female OCD patients concerning specific psychiatric comorbidities, including PTSD, which were related to female, but not to male gender. Similarly, other authors found higher prevalence of OCD in a specific population of veterans.^34,36,48^ It is worth stressing that comorbidity of PTSD with OCD increases the severity of OCD, especially the compulsions.^47^

**Table 4:** Comparison of the main psychiatric comorbidities for PTSD and OCD

	PTSD (%)	OCD (%)
Any psychiatric disorder	37 to 56^78^	90^86^ to 92^79^
		
Any anxiety disorder	35 to 54^78^	76^86^
Generalized anxiety disorder	13 to 15^80^	8^86^ to 35^75^
Panic disorder	11 to 13^80^	13 to 56^94^
Agoraphobia	20^78^	8^86^
Simple phobia	30^786^	32^75^ to 43^86^
Social phobia	10 to 28^80^	19^95^ to 44^86^
Affective disorders	56 to 78^78^	63^86^ to 70^75^
Major depression	48^96^ to 60^78^	28^79^ to 70^75^
Dysthymia	23^78^	3^79^ to 13^86^
Bipolar disorder	16^99^ to 20^100^	10%^75^
Conduct disorder	24^78^	14^86^
Substance use disorder	56 to 78^78^	39^86^
Alcohol abuse/dependence	35 to 52^97^	8^756^to 39^86^
Drug abuse/dependence	19 to 35^97^	2^87^ to 22^86^
Tic disorders	*	36^75^
Separation anxiety	69^98^	25^75^
Any eating disorders	23 to 25^107^	14^75^

OCD = obsessive-compulsive disorder; PTSD = post-traumatic stress disorder.

Based on references.^1,6,10,11,26,43,44,48,69-72,94,95,97-99,107-111^ The prevalence rates were mathematically adjusted.

* No epidemiological data (just case reports).

#### Suicidality

More than 50% of PTSD patients seem to present some lifetime aspect of suicidality: almost 40% report ideation, 9% have planned suicide, and 10% have attempted suicide since the traumatic event.^29,109,112^ Almost one-third attempt suicide more than once.^112^ Although some may conjecture that this could be due to comorbid depression, a 2-year prospective analysis indicated that PTSD only at baseline was predictive of greater risk of suicide ideation and attempts than major depression disorder,^110,111^ and even patients with residual PTSD symptoms or subclinical PTSD presentation seem to have greater risk for suicide ideation.^113^ Rojas et al.^113^ suggested that alcohol dependence was a critical comorbid risk factor for acquiring the capability for suicide attempts. A US study with a large sample (n = 5,877) found that people with PTSD were almost three times more prone to suicidal ideation (odds ratio [OR] = 2.79) and suicide attempts (OR = 2.67).^114^ Although none of the other anxiety disorders were significantly associated with suicidal ideation or attempts, more recent data shows that OCD patients have higher suicidality than found in previous studies. Pellegrini et al.^51^ conducted a systematic review and meta-analysis in which pooled prevalence was 13.5% for suicide attempts, 27.3% for current suicidal ideation, and 47.3% for lifetime suicidal ideation. Severity of obsessions, certain specific obsession content types (e.g., unacceptable thoughts, sexual/religious) comorbid disorders (e.g., substance use, intermittent explosive disorder), depressive/anxious symptoms, and history of suicidality increased the risk,^14,51,115,116^ whereas compulsions had a comparatively protective effect.^51^

Thus, both PTSD and OCD are strongly related to suicidality, although the reasons and risk factors may differ between the two disorders. Some authors seem to relate it to factors such as co-occurrence with major depression (and/or depressive symptoms), but others suggest that suicidality may be a psychological feature independently related to each of the disorders, constituting a transdiagnostic phenomenon.^116^ Since we can calculate a determination coefficient (r^2^), which is the proportion of the variance in the dependent variable that is predictable from the independent variable(s), we could use the results from some authors to try to describe how much of the severity of the disorders may contribute to suicidality severity. For example, as described by Liu et al.^117^ in a sample from China, the correlation between the severities of PTSD and suicide was r = 0.46, indicating that 21% of the severity of PTSD contributes to the severity of suicidality. Similarly, in a sample from India, Dhyani et al.^118^ observed that the correlation between the severities of OCD and suicide was 0.68, which indicates that the severity of OCD may contribute to 46% of the severity of suicidality.

## Final considerations

Since PTSD and OCD are both prevalent and share some psychopathological features (such as ABs, depressive, anxious, and somatic comorbid aspects, suicidality, psychiatric comorbidities, and interference with sexuality, appetite, and sleep patterns), it could be argued that they could share some common neurobiological aspects and belong to the same spectrum of disorders. However, the intersection of their characteristics seems to occur in the extrinsic psychopathological dimension. The core psychopathological symptoms (intrinsic characteristics) are distinctly different, since flashbacks and obsessions are consequences of different predominant defective mental functions: the former derives from defective memory, the latter from defective thought. In the same way, the ABs observed in each disorder are derived from different purposes and inner necessities.

Comorbidity of PTSD and OCD needs further and adequate investigation since post-traumatic OCD with previous OCS and post-traumatic OCD without previous OCS may signal distinct neurobiological and/or genetic aspects.

Some limitations of this scoping review derive from the fact that only one database was used and the search was limited to three publication languages. The scarcity of exploratory psychopathological studies including both disorders (no studies were found that compared PTSD, OCD, and PTSD+OCD groups, for example) restricted the availability of robust evidence to better describe other similar or distinct aspects of these disorders, such as genetic/familial history, neuroimaging results/neurocircuitry, or neurophysiological or neuropsychological examination findings.

## References

[1] American Psychiatric Association (2013). Diagnostic and Statistical Manual of Mental Disorders, Fifth Edition (DSM-5).

[2] Franklin CL, Raines AM (2019). The overlap between OCD and PTSD: Examining self-reported symptom dierentiation. Psychiatry Res.

[3] Green B (2003). Post-traumatic stress disorder: symptom profiles in men and women. Curr Med Res Opin.

[4] McKeon J, Roa B, Mann A (1984). Life events and personality traits in obsessive–compulsive neurosis. Br J Psychiatry.

[5] Imthon AK, Caldart CA, do Rosário MC, Fontenelle LF, Miguel EC, Ferrão IA (2002). Stressful life events and the clinical expression of Obsessive-Compulsive Disorder (OCD): an exploratory study. J Clin Med.

[6] Auxéméry Y (2018). Post-traumatic psychiatric disorders: PTSD is not the only diagnosis. Presse Med.

[7] Frederick JW (2003). Freud’s case of the Rat Man revisited: an existential-phenomenological and socio-historical analysis. J Phenomenal Psycho.

[8] Lafleur DL, Petty C, Mancuso E, McCarthy K, Biederman J, Faro A (2011). Traumatic events and obsessive compulsive disorder in children and adolescents: is there a link?. J Anxiety Disord.

[9] Morina N, Sulaj V, Schnyder U, Klaghofer R, Müller J, Martin-Sölch C (2016). Obsessive-compulsive and posttraumatic stress symptoms among civilian survivors of war. BMC Psychiatry.

[10] Fontenelle LF, Cocchi L, Harrison BJ, Shavitt RG, do Rosário MC, Ferrão YA (2012). Towards a post-traumatic subtype of obsessive–compulsive disorder. J Anxiety Disord.

[11] Grabe HJ, Ruhrmann S, Spitzer C, Josepeit J, Ettelt S, Buhtz F (2008). Obsessive-compulsive disorder and posttraumatic stress disorder. Psychopathology.

[12] Gershuny BS, Baer L, Parker H, Gentes EL, Infield AL, Jenike MA (2008). Trauma and posttraumatic stress disorder in treatment-resistant obsessive-compulsive disorder. Depress Anxiety.

[13] Avila RCS, do Nascimento LG, Porto RLM, Fontenelle L, Filho ECM, Brakoulias V (2019). Level of insight in patients with obsessive-compulsive disorder: an exploratory comparative study between patients with “Good Insight” and “Poor Insight”. Front Psychiatry.

[14] Torres AR, Ramos-Cerqueira ATA, Ferrão YA, Fontenelle LF, do Rosário MC (2011). Suicidality in obsessive-compulsive disorder: prevalence and relation to symptom dimensions and comorbid conditions. J Clin Psychiatry.

[15] Ay R, Erbay LG (2018). Relationship between childhood trauma and suicide probability in obsessive-compulsive disorder. Psychiatry Res.

[16] Khosravani V, Kamali Z, Jamaari Ardakani R, Samimi Ardestani M (2017). The relation of childhood trauma to suicide ideation in patients suffering from obsessive-compulsive disorder with lifetime suicide attempts. Psychiatry Res.

[17] Barzilay R, Patrick A, Calkins ME, Moore TM, Gur RC, Gur RE (2019). Association between early-life trauma and obsessive compulsive symptoms in community youth. Depress Anxiety.

[18] Semiz UB, Inanc L, Bezgin CH (2014). Are trauma and dissociation related to treatment resistance in patients with obsessive-compulsive disorder?. Soc Psychiatry Psychiatr Epidemiol.

[19] Lochner C, Seedat S, Hemmings SM, Kinnear CJ, Corfield VA, Niehaus DJ (2004). Dissociative experiences in obsessive-compulsive disorder and trichotillomania: clinical and genetic findings. Compr Psychiatry.

[20] Lochner C, Seedat S, Hemmings SM, Moolman-Smook JC, Kidd M, Stein DJ (2007). Investigating the possible effects of trauma experiences and 5-HTT on the dissociative experiences of patients with OCD using path analysis and multiple regression. Neuropsychobiology.

[21] Fontenelle LF, Domingues AM, Souza WF, Mendlowicz MV, Menezes GB, Figueira IL (2007). History of trauma and dissociative symptoms among patients with obsessive-compulsive disorder and social anxiety disorder. Psychiatr Q.

[22] Shavitt RG, Valério C, Fossaluza V, Silva EM, Cordeiro Q, Diniz JB (2010). The impact of trauma and post-traumatic stress disorder on the treatment response of patients with obsessive-compulsive disorder. Eur Arch Psychiatry Clin Neurosci.

[23] Galvão TF, Pansani TSA, Harrad D (2015). Principais itens para relatar revisões sistemáticas e meta-análises: a recomendação PRISMA. Epidemiol Serv Saude.

[24] Afari N, Ahumada SM, Wright LJ, Mostoufi S, Golnari G, Reis V (2014). Psychological trauma and functional somatic syndromes: a systematic review and meta-analysis. Psychosom Med.

[25] Avdibegovic E, Delic A, Hadzibeganovic K, Selimbasic Z (2010). Somatic diseases in patients with posttraumatic stress disorder. Medicinski Arhiv.

[26] Badour CL, Bown S, Adams TG, Bunaciu L, Feldner MT (2012). Specificity of fear and disgust experienced during traumatic interpersonal victimization in predicting posttraumatic stress and contamination-based obsessive-compulsive symptoms. J Anxiety Disord.

[27] Bond S, Gourlay C, Desjardins A, Bodson-Clermont P, Boucher ME (2017). Anxiety, depression and PTSD-related symptoms in spouses and close relatives of burn survivors: when the supporter needs to be supported. Burns.

[28] Brady KT, Killeen TK, Brewerton T, Lucerini S (2000). Comorbidity of psychiatric disorders and posttraumatic stress disorder. J Clin Psychiatry.

[29] Brakoulias V, Starcevic V, Belloch A, Brown C, Ferrao YA, Fontenelle LF (2017). Comorbidity, age of onset and suicidality in obsessive–compulsive disorder (OCD): an international collaboration. Compr Psychiatry.

[30] Brewin CR (2014). Episodic memory, perceptual memory, and their interaction: foundations for a theory of posttraumatic stress disorder. Psychol Bull.

[31] Brewin CR (2015). Re-experiencing traumatic events in PTSD: new avenues in research on intrusive memories and flashbacks. Eur J Psychotraumatol.

[32] Ferrão YA, Shavitt RG, Prado H, Fontenelle LF, Malavazzi DM, Mathis MA (2012). Sensory phenomena associated with repetitive behaviors in obsessive-compulsive disorder: an exploratory study of 1001 patients. Psychiatry Res.

[33] Forbes D, Fletcher S, Lockwood E, O’Donnell M, Creamer M, Bryant RA (2011). Requiring both avoidance and emotional numbing in DSM-V PTSD: will it help?. J Affect Disord.

[34] Fostick L, Nacasch N, Zohar J (2012). Acute obsessive compulsive disorder (OCD) in veterans with posttraumatic stress disorder (PTSD). World J Biol Psychiatry.

[35] Gershuny BS, Baer L, Jenike MA, Minichiello WE, Wilhelm S (2002). Comorbid posttraumatic stress disorder: impact on treatment outcome for obsessive-compulsive disorder. Am J Psychiatry.

[36] Gros DF, Magruder KM, Frueh BC (2013). Obsessive compulsive disorder in veterans in primary care: prevalence and impairment. Gen Hosp Psychiatry.

[37] Huppert JD, Moser JS, Gershuny BS, Riggs DS, Spokas M, Filip J (2005). The relationship between obsessive- compulsive and posttraumatic stress symptoms in clinical and non-clinical samples. J Anxiety Disord.

[38] Iverach L, Menzies RG, Menzies RE (2014). Death anxiety and its role in psychopathology: reviewing the status of a transdiagnostic construct. Clin Psychol Rev.

[39] Kessler RC, Sonnega A, Bromet E, Hughes M, Nelson CB (1995). Posttraumatic stress disorder in the National Comorbidity Survey. Arch Gen Psychiatry.

[40] Kroska EB, Miller ML, Roche AI, Kroska SK, O’Hara MW (2018). Effects of traumatic experiences on obsessive-compulsive and internalizing symptoms: The role of avoidance and mindfulness. J Affect Disord.

[41] Liu L, Wang L, Cao C, Cao X, Zhu Y, Liu P (2018). Serotonin transporter 5-HTTLPR genotype is associated with intrusion and avoidance symptoms of DSM-5 posttraumatic stress disorder (PTSD) in Chinese earthquake survivors. Anxiety Stress Coping.

[42] Mataix-Cols D, Pertusa A, Leckman JF (2007). Issues for DSM-V: how should obsessive-compulsive and related disorders be classified?. Am J Psychiatry.

[43] Menzies RE, Dar-Nimrod I (2017). Death anxiety and its relationship with obsessive-compulsive disorder. J Abnorm Psychol.

[44] Menzies RE, Zuccala M, Sharpe L, Dar-Nimrod I (2021). Are anxiety disorders a pathway to obsessive-compulsive disorder? Different trajectories of OCD and the role of death anxiety. Nord J Psychiatry.

[45] Menzies RE, Zuccala M, Sharpe L, Dar-Nimrod I (2020). Subtypes of obsessive compulsive disorder and their relationship to death anxiety. J Obsessive Compuls Relat Disord.

[46] Merrill A, Gershuny B, Baer L, Jenike MA (2011). Depression in comorbid obsessive-compulsive disorder and posttraumatic stress disorder. J Clin Psychol.

[47] Miller ML, Brock RL (2017). The effect of trauma on the severity of obsessive-compulsive spectrum symptoms: a meta-analysis. J Anxiety Disord.

[48] Nacasch N, Fostick L, Zohar J (2011). High prevalence of obsessive-compulsive disorder among posttraumatic stress disorder patients. Eur Neuropsychopharmacol.

[49] Nissen JB, Parner E (2018). The importance of insight, avoidance behavior, not-just-right perception and personality traits in pediatric obsessive-compulsive disorder (OCD): a naturalistic clinical study. Nord J Psychiatry.

[50] Ojserkis R, Boisseau CL, Reddy MK, Mancebo MC, Eisen JL, Rasmussen AS (2017). The impact of lifetime PTSD on the seven-year course and clinical characteristics of OCD. Psychiatry Res.

[51] Pellegrini P, Maietti E, Rucci P, Casadei G, Maina G, Fineberg N (2002). Suicide attempts and suicidal ideation in patients with obsessive-compulsive disorder: a systematic review and meta-analysis. J Affect Disord.

[52] Richards A, Kanady JC, Neylan TC (2020). Sleep disturbance in PTSD and other anxiety-related disorders: an updated review of clinical features, physiological characteristics, and psychological and neurobiological mechanisms. Neuropsychopharmacology.

[53] Ruscio AM, Stein DJ, Chiu WT, Kessler RC (2010). The epidemiology of obsessive-compulsive disorder in the National Comorbidity Survey Replication. Mol Psychiatry.

[54] Sikharulidze G, van Geloven N, Lelashvili E, Kalandarishvili G, Gugushvili N, Vermetten E (2017). Posttraumatic stress disorder and somatic complaints in a deployed cohort of Georgian Military personnel: mediating effect of depression and anxiety. J Trauma Stress.

[55] Stein DJ, Fineberg NA, Bienvenu OJ, Denys D, Lochner C, Nestadt G (2010). Should OCD be classified as an anxiety disorder in DSM-V?. Depress Anxiety.

[56] Torres AR, Shavitt RG, Torresan RC, Ferrão YA, Miguel EC, Fontenelle LF (2013). Clinical features of pure obsessive-compulsive disorder. Compr Psychiatry.

[57] Torresan RC, Ramos-Cerqueira AT, Shavitt RG, do Rosário MC, Mathis MA, Miguel EC (2013). Symptom dimensions, clinical course and comorbidity in men and women with obsessive-compulsive disorder. Psychiatry Res.

[58] Unseld M, Krammer K, Lubowitzki S, Jachs M, Baumann L, Vyssoki B (2019). Screening for post-traumatic stress disorders in 1017 cancer patients and correlation with anxiety, depression, and distress. Psychooncology.

[59] Valderrama J, Hansen SK, Pato C, Phillips K, Knowles J, Pato MT (2020). Greater history of traumatic event exposure and PTSD associated with comorbid body dysmorphic disorder in a large OCD cohort. Psychiatry Res.

[60] Wheaton MG, Gershkovich M, Gallagher T, Foa EB, Simpson HB (2018). Behavioral avoidance predicts treatment outcome with exposure and response prevention for obsessive-compulsive disorder. Depress Anxiety.

[61] Van der Kolk BA, Van der Hart O (1989). Pierre Janet and the breakdown of adaptation in psychological trauma. Am J Psychiatry.

[62] World Health Organization (WHO) (2018). International classification of diseases for mortality and morbidity statistics. 11th revision.

[63] Andrewes DG, Jenkins LM (2019). The role of the amygdala and the ventromedial prefrontal cortex in emotional regulation: implications for post-traumatic stress disorder. Neuropsychol Rev.

[64] Rachman S (1998). A cognitive theory of obsessions: elaborations. Behav Res Ther.

[65] Van de Heuvel OA, Van Wingen G, Soriano-Mas C, Alonso P, Chamberlain SR, Nakamae T (2016). Brain circuitry of compulsivity. Eur Neuropsychopharmacol.

[66] Shephard E, Stern ER, van den Heuvel OA, Costa DLC, Batistuzzo MC, Godoy PBG (2021). Toward a neurocircuit-based taxonomy to guide treatment of obsessive-compulsive disorder. Mol Psychiatry.

[67] Aristides VC (2014). O TOC e suas manifestações. Manual de terapia cognitivo-comportamental para o transtorno obsessivo-compulsivo.

[68] Mowrer OH (1939). A stimulus-response theory of anxiety. Psychol Rev.

[69] Aaseth J, Roer GE, Lien L, Bjørklund G (2019). Is there a relationship between PTSD and complicated obesity? A review of the literature. Biomed Pharmacother.

[70] Mazereel V, Detraux J, Vancampfort D, van Winkel R, De Hert M (2020). Impact of psychotropic medication effects on obesity and the metabolic syndrome in people with serious mental illness. Front Endocrinol (Lausanne).

[71] Fawcett E, Power H, Fawcett JM (2020). Women are at greater risk of OCD than men: a meta-analytic review of OCD prevalence worldwide. J Clin Psychiatry.

[72] Alegria M, Jackson JS, Kessler RC, Takeuchi D (2016). Collaborative Psychiatric Epidemiology Surveys (CPES), 2001-2003 [United States] (ICPSR 20240).

[73] Miguel EC, Ferrão YA, Rosário MC, Mathis MA, Torres AR, Fontenelle LF (2008). The Brazilian Research Consortium on Obsessive-Compulsive Spectrum Disorders: recruitment, assessment instruments, methods for the development of multicenter collaborative studies and preliminary results. Braz J Psychiatry.

[74] Stein DJ, Costa DLC, Lochner C, Miguel EC, Janardhan Reddy YC, Shavitt RG (2019). Obsessive-compulsive disorder. Nat Rev Dis Primers.

[75] Otte C (2008). Incomplete remission in depression: role of psychiatric and somatic comorbidity. Dialogues Clin Neurosci.

[76] Lee K, Kim D, Cho Y (2018). Exploratory factor analysis of the Beck Anxiety Inventory and the Beck Depression Inventory-II in a psychiatric outpatient population. J Korean Med Sci.

[77] Pozza A, Veale D, Marazziti D, Delgadillo J, Albert U, Grassi G (2020). Sexual dysfunction and satisfaction in obsessive compulsive disorder: protocol for a systematic review and meta-analysis. Syst Rev.

[78] Burri A, Spector T (2011). Recent and lifelong sexual dysfunction in a female UK population sample: prevalence and risk factors. J Sex Med.

[79] Kendurkar A, Kaur B (2008). Major depressive disorder, obsessive-compulsive disorder, and generalized anxiety disorder: do the sexual dysfunctions differ? Prim Care Companion. J Clin Psychiatry.

[80] Pozza A, Marazziti D, Mucci F, Grassi G, Prestia D, Dèttore D (2020). Sexual arousal in obsessive-compulsive disorder with and without contamination/washing symptoms: a moderating role of disgust sensitivity. J Nerv Ment Dis.

[81] Pavlov IP (1938). Conditioned reflexes.

[82] Skinner BF (1937). Two types of conditioned reflex: a reply to Konorski and Miller. J Gen Psychol.

[83] Skinner BF (1938). O Comportamento dos organismos: uma análise experimental.

[84] Pigott TA, l`Heureux F, Dubbert B, Bernstein S, Murphy DL (1994). Obsessive compulsive disorder: comorbid conditions. J Clin Psychiatry.

[85] Robins LN, Helzer JE, Weissman MM, Orvaschel H, Gruenberg E, Burke JD (1984). Lifetime prevalence of specific psychiatric disorders in three sites. Arch Gen Osychiatry.

[86] Herman JL (1992). Complex PTSD: a syndrome in survivors of prolonged and repeated trauma. J Trauma Stress.

[87] Pochard F, Azoulay E, Chevret S, Lemaire F, Hubert P, Canoui P (2001). Symptoms of anxiety and depression in family members of intensive care unit patients: ethical hypothesis regarding decision-making capacity. Crit Care Med.

[88] Jones M, Krochmalik A (2003). Obsessive-compulsive disorder: theory, research and treatment.

[89] Vaccaro LD, Vaccaro MK, Menzies RG, Tl St Clare (2010). Danger ideation reduction therapy (DIRT) for obsessive-compulsive checkers: a comprehensive guide to treatment.

[90] Mehlum L, Weisaeth L (2002). Predictors of posttraumatic stress reactions in Norwegian U.N. peacekeepers 7 years after service. J Trauma Stress.

[91] Bosco MA, Gallinati JL, Clark ME (2013). Conceptualizing and treating comorbid chronic pain and PTSD. Pain Res Treat.

[92] Miguel EC, Rosario-Campos MCD, Prado HDS, Valle RD, Rauch SL, Coffey BJ (2000). Sensory phenomena in obsessive-compulsive disorder and Tourette’s disorder. J Clin Psychiatry.

[93] Feske U, Frank E, Kupfer DJ, Shear MK, Weaver E (1998). Anxiety as a predictor of response to interpersonal psychotherapy for recurrent major depression: an exploratory investigation. Depression Anxiety.

[94] Ghassemzadeh H, Raisi F, Firoozikhojastefar R, Meysamie A, Karamghadiri N, Nasehi AA (2017). A study on sexual function in Obsessive-Compulsive Disorder (OCD) patients with and without depressive symptoms. Perspect Psychiatr Care.

[95] Fuss J, Briken P, Stein DJ, Lochner C (2019). Compulsive sexual behavior disorder in obsessive-compulsive disorder: prevalence and associated comorbidity. J Behav Addict.

[96] Cosgrove DJ, Gordon Z, Bernie JE, Hami S, Montoya D, Stein MB (2002). Sexual dysfunction in combat veterans with post-traumatic stress disorder. Urology.

[97] Yehuda R, Lehrner A, Rosenbaum TY (2015). PTSD and sexual dysfunction in men and women. J Sex Med.

[98] DiMauro J, Renshaw KD (2019). PTSD and relationship satisfaction in female survivors of sexual assault. Psychol Trauma.

[99] Abramovitch A, Anholt EG, Cooperman A, van Balkom JLMA, Giltay EJ, Penninx BW (2019). Body mass index in obsessive-compulsive disorder. J Affect Disord.

[100] Hall A, Kimberly A, Bartlett BA, Iverson KM, Mitchell KS (2017). Military-related trauma is associated with eating disorder symptoms in male veterans. Int J Eat Disord.

[101] Koren D, Arnon I, Lavie P, Klein E (2002). Sleep complaints as early predictors of posttraumatic stress disorder: a 1-year prospective study of injured survivors of motor vehicle accidents. Am J Psychiatry.

[102] Gehrman P, Ad Seelig, Jacobson IG, Boyko EJ, Hooper TI, Gackstetter GD (2013). Predeployment sleep duration and insomnia symptoms as risk factors for new-onset mental health disorders following military deployment. Sleep.

[103] Van Liempt S, Van Zuiden M, Westenberg H, Super A, Vermetten E (2013). Impact of impaired sleep on the development of PTSD symptoms in combat veterans: a prospective longitudinal cohort study. Depress Anxiety.

[104] Paterson JL, Reynolds AC, Ferguson SA, Dawson D (2013). Sleep and obsessive-compulsive disorder (OCD). Sleep Med Rev.

[105] Haack M, Mullington JM (2005). Sustained sleep restriction reduces emotional and physical well-being. Pain.

[106] Kessler RC (2008). National comorbidity survey: baseline (NCS-1), 1990-1992.

[107] Mitchell K, Wolf E, Lyons M, Goldberg J, Magruder K (2018). A co-twin control study of the association between combat exposure, PTSD and obesity in male veterans. Psychol Med.

[108] Pagoto SL, Schneider KL, Bodenlos JS, Appelhans BM, Whited MC, Ma Y (2012). Association of post-traumatic stress disorder and obesity in a nationally representative sample. Obesity (Silver Spring).

[109] Wilcox HC, Storr CL, Breslau N (2009). Posttraumatic stress disorder and suicide attempts in a community sample of urban American young adults. Arch Gen Psychiatry.

[110] Cougle JR, Resnick H, Kilpatrick DG (2009). PTSD, depression, and their comorbidity in relation to suicidality: cross-sectional and prospective analyses of a national probability sample of women. Depress. Anxiety.

[111] Marshall RD, Olfson M, Hellman F, Blanco C, Guardino M, Struening EL (2001). Comorbidity, impairment, and suicidality in subthreshold PTSD. Am J Psychiatry.

[112] Tarrier N, Gregg L (2004). Suicide risk in civilian PTSD patients. Soc Psychiatry Psychiatr Epidemiol.

[113] Rojas SM, Bujarski S, Babson KA, Dutton CE, Feldner M (2014). Understanding PTSD comorbidity and suicidal behavior: associations among histories of alcohol dependence, major depressive disorder, and suicidal ideation and attempts. J Anxiety Disord.

[114] Sareen J, Houlahan T, Cox BJ, Asmundson G (2005). Anxiety disorders associated with suicidal ideation and suicide attempts in the national comorbidity survey. J Nerv Ment Dis.

[115] Albert U, De Ronchi D, Maina G, Pompili M (2019). Suicide risk in obsessive-compulsive disorder and exploration of risk factors: a systematic review. Curr Neuropharmacol.

[116] Agne N, Tisott C, Ballester P, Passos I, Ferrão YA (2021). Predictors of suicide attempt in patients with obsessive-compulsive disorder: an exploratory study with machine learning analysis. Psychol Med.

[117] Liu A, Wang W, Wu X (2020). Understanding the relation between self-compassion and suicide risk among adolescents in a post-disaster context: mediating roles of gratitude and posttraumatic stress disorder. Front Psychol.

[118] Dhyani M, Trivedi JK, Nischal A, Sinha PK, Verma S (2013). Suicidal behaviour of Indian patients with obsessive compulsive disorder. Indian J Psychiatry.

