# Supplementary material for: Psychopathological intersection between obsessive-compulsive disorder and post-traumatic stress disorder: scoping review of similarities and differences
Source: Trends Psychiatry Psychother. 2023 May 19;45:e20210370. doi: 10.47626/2237-6089-2021-0370 (PMC10241527; doi:10.47626/2237-6089-2021-0370)
Supplement: Supplementary file 1 [file 2238-0019-trends-45-e20210370-suppl.pdf]

http://doi.org/10.47626/2237-6089-2021-0370

Table S1 - Detailed description of the search results according to the different search strategies.

		Number of studies excluded according to the selection criteria					
**Strategy**	**Screened**	**Language criteria**	**Not human subject**	**Not journal articles**	**No abstract available**	**Case reports**	**Total Records**
OCD x PTSD	821	63	115	10	19	22	592
OCD x Trauma	555	44	82	32	27	88	282
Obsession x PTSD	772	48	120	6	5	22	571
Obsession x trauma	622	49	104	18	12	87	352
Obsession x flashback	9	2	2	0	0	1	4
OCD x flashback	3	1	0	0	0	1	1

OCD = obsessive-compulsive disorder; PTSD = post-traumatic stress disorder.
